# Characterization of a novel thermostable and xylose-tolerant GH 39 β-xylosidase from *Dictyoglomus thermophilum*

**DOI:** 10.1186/s12896-018-0440-3

**Published:** 2018-05-21

**Authors:** Qi Li, Tao Wu, Zhipeng Qi, Linguo Zhao, Jianjun Pei, Feng Tang

**Affiliations:** 1 Co-Innovation Center for Sustainable Forestry in Southern China, Nanjing Forestry University, 159 Long Pan Road, Nanjing, 210037 China; 2 College of Chemical Engineering, Nanjing Forestry University, 159 Long Pan Road, Nanjing, 210037 China; 3 Jiangsu Key Lab for the Chemistry & Utilization of Agricultural and Forest Biomass, 159 Long Pan Road, Nanjing, 210037 China; 4 International Centre for Bamboo and Rattan, 8 Fu Tong East Street, Beijing, 100714 China

**Keywords:** *Dictyoglomus thermophilum*, β-xylosidase, Xylose tolerant, Xylooligosaccharides, Biotransformation

## Abstract

**Background:**

β-D-xylosidase is a vital exoglycosidase with the ability to hydrolyze xylooligosaccharides to xylose and to biotransform some saponins by cleaving outer β-xylose. β-D-xylosidase is widely used as one of the xylanolytic enzymes in a diverse range of applications, such as fuel, food and the pharmaceutical industry; therefore, more and more studies have focused on the thermostable and xylose-tolerant β-D-xylosidases.

**Results:**

A thermostable β-xylosidase gene (*xln-DT*) of 1509 bp was cloned from *Dictyoglomus thermophilum* and expressed in *E.coli* BL21. According to the amino acid and phylogeny analyses, the β-xylosidase Xln-DT is a novel β-xylosidase of the GH family 39. The recombinant β-xylosidase was purified, showing unique bands on SDS-PAGE, and had a protein molecular weight of 58.7 kDa. The β-xylosidase Xln-DT showed an optimal activity at pH 6.0 and 75 °C, with p-nitrophenyl-β-D-xylopyranoside (pNPX) as a substrate. Xln-DT displayed stability over a pH range of 4.0-7.5 for 24 h and displayed thermotolerance below 85 °C. The values of the kinetic parameters *K*_*m*_ and *V*_*max*_ for pNPX were 1.66 mM and 78.46 U/mg, respectively. In particular, Xln-DT displayed high tolerance to xylose, with 60% activity in the presence of 3 M xylose. Xln-DT showed significant effects on the hydrolyzation of xylobiose. After 3 h, all the xylobiose tested was degraded into xylose. Moreover, β-xylosidase Xln-DT had a high selectivity for cleaving the outer xylose moieties of natural saponins, such as notoginsenoside R1 and astragaloside IV, which produced the ginsenoside Rg1 with stronger anti-fatigue activity and produced cycloastragenol with stronger anti-aging activity, respectively.

**Conclusion:**

This study provides a novel GH 39 β-xylosidase displaying extraordinary properties of highly catalytic activity at temperatures above 75 °C, remarkable hydrolyzing activity of xylooligosaccharides and rare saponins producing ability in the pharmaceutical and commercial industries.

**Electronic supplementary material:**

The online version of this article (10.1186/s12896-018-0440-3) contains supplementary material, which is available to authorized users.

## Background

Recently, more and more studies have focused on cellulose and hemicellulose hydrolysis in an effort to use lignocellulosic residues as feedstock for producing bioethanol [1, 2]. The plant cell wall mainly consists of 30-45% cellulose, 20-30% hemicellulose and 5-20% lignin [3]. Hemicelluloses, as the second most abundant renewable lignocellulosic biomass resource, are a series of heteropolysaccharides that contain xylans, arabinans, glucans and mannans, in which xylan is the major hemicellulosic polysaccharide that can be efficiently hydrolyzed into its sugar constituents [4–6]. The xylan backbone consists of β-(1,4)-linked xylopyranosyl units with side-chains, such as acetate, glucuronosyl and arabinofuranosyl depending on the source. Complete xylan degradation requires the concerted action of xylanolytic enzymes, owing to the complex structure. First, xylanase randomly cleaves the β-1,4 bonds in the xylan to yield xylooligosaccharides (XOs), xylobiose and xylose [7]. Second, α-arabinofuranosidase, acetylesterase, α-glucuronidase and ferulic acid esterase cleave the side-chain substituents [8]. In the last step, β-xylosidase, which is well known as the major hydrolytic enzyme that degrades the non-reducing ends of β-1,4-linked D-xylose residues to release xylose as the final product from short xylooligosaccharides [9, 10]. However, the roles of β-xylosidase include, but are not limited to, the degradation of xylooligosaccharide. β-xylosidase has large potential in many biotechnological applications, especially in the fuel and food industries as well as the bioconversion of saponins [11–13].

Saponin is a kind of bioactive substance with a range of physiological functions, such as anti-inflammatory, anti-cancer, immunoregulatory and anti-fatigue effects [14, 15]. Various saponins have disparate physiological activities as a result of diverse skeletons and sugar moieties. Thus, many studies have concentrated more on biotransforming glycosylated saponins to deglycosylated saponins by removing the sugar moieties with enzymes, such as β-glucosidase and α-rhamnosidase, to achieve better pharmacological effects [16, 17]. Furthermore, part of the saponins are needed to hydrolyze the xylose moiety by β-xylosidases. Unfortunately, until recently, it has been difficult to find a highly thermostable recombinant β-xylosidase with the ability to convert saponins. Ginsenoside Rg1 (Rg1) and cycloastragenol (CA) are the major bioactive constituents in the plants *Panax notoginseng* and *Astragali radix*, respectively [18, 19], which have been considered to be remarkable ingredients with pharmacological activities [20, 21]. Chinese Pharmacopoeia (Edition 2015) has used Rg1 as the major index component for the Quality Control (QC) of ginseng herbs [22]. However, it is quite difficult to improve the purity of the exact quantification of Rg1 because there are many homologous components with highly similar physicochemical properties from the target compound. Therefore, conversion from notoginsenoside R1 to Rg1 can greatly improve the purity of Rg1 [12]. CA, as the aglycone of astragaloside IV (ASI), can activate telomerase, which displays great potential for anti-aging activity [23]. Nevertheless, it is very difficult to isolate CA from *Astragali radix* due to its low concentration. Therefore, conversion from ASI to CA could increase the content of CA efficiently. Methods such as acid hydrolysis, heating treatment, steaming treatment, and microbial and enzymatic transformation, have been used to convert these saponins [24–26]. Among these methods, enzymatic transformation with β-xylosidase has exhibited potential because of its mild reaction condition and high specificity.

As is well known, β-xylosidases are largely found in GH families 1, 3, 30, 39, 43, 52, 54 and 120, according to the GH classification system CAZy (Carbonhydrate-Active Enzymes Database) [27]. Until recently, many β-xylosidases from different GH families have been described in a variety of microorganisms including bacteria, archaea, fungi and plant [28–31]. Generally, xylosidases from fungal sources have optimum pH values from 3 to 5 and an optimum temperature range from 40 °C to 60 °C, thus far, these xylosidases have only been grouped into GH 3, GH 43 and GH 54 [32, 33]. Literature about the extremely thermophilic and xylose-tolerant β-xylosidases is relatively scarce. Therefore, thermophilic bacteria are regarded as a remarkable source of thermotolerant β-xylosidases [34–36]. Usually, β-xylosidases with thermostability, greater specific activity and a greater tolerance to xylose are used in industrial processes, since high temperatures increase the solubility of substrates and reduce the risk of contamination, and the resistance to xylose can reduce the inhibition of substrate feedback [37]. As a result, the search for thermostable and xylose-tolerant β-xylosidases has increased considerably in recent years.

In this article, we cloned, expressed and characterized a novel thermostable GH 39 β-xylosidase from *Dictyoglomus thermophilum* DSM 3960. In particular, the high tolerance to xylose makes this β-xylosidase useful for various biotechnological applications. Moreover, this enzyme exhibited highly selective hydrolysis for outer xylose in notoginsenoside R1 and CA. These extraordinary properties make Xln-DT more suitable for producing rare saponins in the pharmaceutical and commercial industries.

## Methods

### Bacterial strains, plasmids and materials

*Dictyoglomus thermophilum* DSM 3960 was purchased from DSMZ (www.dsmz.de). The *E. coli* BL21 (DE3) and Top10F’ cells were grown in LB broth at 37 °C containing 100 mg/ml ampicillin. The vector pET-20b (Novagen, USA) was employed as a cloning and expression vector. The β-xylosidases XlnD, Tth XyB3, Tth Xyl and Tpe Xln3 and the β-glucosidase Tpebg3 were prepared by the Microbial Technology Research Laboratory, Nanjing Forestry University.

The substrates *p*NPX, *p*NP-β-D-glucopyranoside, *p*NP-β-D-galactopyranoside, *p*NPR, *p*NPAF and *p*NPAP were purchased from Sigma (USA). The sugar xylobiose was purchased from Aladdin (China). Notoginsenoside R1 (> 98% Purity, HPLC), ginsenoside Rg1 (> 98% Purity, HPLC), ASI (> 98% Purity, HPLC) and CA (> 98% Purity, HPLC) were purchased from the Chendu Institute of Biology, CAS (www.cdmust.com).

### DNA manipulation

The DNA was manipulated following standard operating procedures. The Gel Extraction Kit and Plasmid Kit (Qiagen, USA) were used for purifying the PCR products and plasmids. The DNA restriction endonucleases *BamH*I and *Xho*I, the T4 DNA ligase and the Ex-Taq restriction polymerase were purchased from TaKaRa (China). The DNA transformation was manipulated by electroporation using a Bio-Rad Gene Pulser (USA).

### Plasmid constructions

The β-xylosidase encoded gene *xln-DT* (accession number: KX449145) with a size of approximately 1500 bp was amplified from *Dictyoglomus thermophilum* DSM 3960 genomic DNA by PCR with the primers *xln-DT*-f (CGCGGATCCATGAACCATATAAAGATTGAAA) and *xln-DT*-r (CCGCTCGAGATATCCACCTGGTATTTTGCTATC). The restriction enzyme sites were the underlined sequences. The amplified PCR products were then digested with *BamH*I and *Xho*I endonucleases and subcloned into the pET-20b vector; finally, the expression plasmid pET-20b-*xln-DT* was obtained.

### Nucleotide sequence analysis of *xln-DT*

The nucleotide sequences of *xln-DT* and the other known β-xylosidase genes were analyzed by the software Clustal 2.1. Databases were searched by using BLAST (http://www.blast.ncbi.nlm.nih.gov/Blast.cgi) at NCBI and against CAZy (www.cazy.org). Then, the neighbor-joining (NJ) evolutionary tree was constructed by the software mega 7.0.

### Expression and purification of Xln-DT

The recombinant plasmid, pET-20b-*xln-DT,* was transformed into *E.coli* BL21 (DE3), grown in LB broth containing 100 mg/ml ampicillin at 37 °C and induced to express recombinant Xln-DT by adding IPTG to a final concentration of 0.1 mM and to an optical density, OD_600_, close to 0.8; the bacteria were further incubated at 28 °C for approximately 8 h.

The recombinant cells (one liter) were harvested by centrifugation at 8, 000×*g* (4 °C) for 20 min, washed with distilled water several times and resuspended in 50 mL 1 × binding buffer (pH 7.9). After sonication, the cell extracts were heat treated at 75 °C for 30 min, cooled in an ice-bath and centrifuged at 10, 000×g (4 °C) for 30 min. Finally, the supernatants were purified with an immobilized metal affinity column (Novagen, USA), and the enzyme protein was collected by eluting in 1 × binding buffer (pH 7.9). The purity of the target protein was canvassed by an SDS-PAGE gel, and the protein bands were analyzed by density scanning with an image analysis system (Bio-Rad, USA) [38]. The purified protein concentration was measured by the Bradford method using albumin from bovine serum as a standard.

### β-Xylosidase assay

The substrate *p*NPX was used for the β-xylosidase activity assay. The reaction mixture was contained with 10 μL of 20 mM substrate *p*NPX, 180 μL of sodium phosphate buffer (50 mM, pH 6.0) and 10 μL of purified enzyme (0.64 μg). After 10 min (75 °C), the reaction was stopped by the addition of 600 μL Na_2_CO_3_ (1 M) [39]. The absorbance of the mixture was immediately measured at 405 nm. The definition of one unit of β-xylosidase activity (1 U) was consistent with the literature reference [36]. For every sample, the activity was measured three separate times.

The impact of temperature was measured by a standard assay over a range of temperatures (60-90 °C) in sodium phosphate buffer (50 mM, pH 6.0). The optimum pH was evaluated by incubation at 75 °C for 10 min in 50 mM sodium phosphate buffers with various pH values (pH 4.0-7.0). For the thermal stability analysis, the residual β-xylosidase activity was determined after the pre-incubation of enzymes (0.64 μg) at a range of temperatures from 65 to 95 °C for 2 h (every 30 min). The activity of the enzyme without pre-incubation was defined as 100%. The pH stability of the enzyme was measured by determining the remaining activity after incubating the enzyme (0.64 μg) in sodium phosphate buffer (50 mM) from pH 4.0 to 7.0 at 4 °C. After 24 h, the residual activity of the enzyme incubated at various pH values was measured. The purified enzyme (0.64 μg) was incubated at temperatures ranging from 75 to 95 °C in sodium phosphate buffer (50 mM, pH 6.0). The half-life was calculated from the first-order rate constants of inactivation, which were obtained from linear regressions in logarithmic coordinates [40]. The influence of various xylose concentrations (250, 500, 750, 1000, 1500, 2000, 2500 and 3000 mM) on the β-xylosidase activity was measured.

The effects of adding different ions and chemical reagents on the β-xylosidase activity of the purified enzyme (0.64 μg) were examined by adding different metal ions into the aforementioned buffer. Ni^2+^, Fe^3+^, Mn^2+^, Mg^2+^, Ca^2+^, K^+^, Zn^2+^, Al^3+^, Li^2+^, Na^+^, NH_4_^+^, Cu^2+^, Fe^2+^, Ba^2+^, Co^2+^, and Hg^2+^ as well as the chemical reagents EDTA and PMSF were assayed at 1 mM and 5 mM of the final concentrations in the reaction mixture.

The effects of organic solvents on the β-xylosidase activity of the purified enzyme (0.64 μg) were measured by adding 5, 10, 15, 20, 25 and 30% organic solvents (methanol, ethanol or DMSO) to the mixture. The β-xylosidase enzyme Xln-DT was mixed with each solvent for 30 min at 75 °C before adding *p*NPX to initiate the enzyme reaction. The activity of the enzyme without the metal ions, chemical reagents or organic solvents was defined as 100%. Every experiment was performed in triplicate.

The substrate specificity of the purified enzyme (0.64 μg) was tested by using *p*NPX, *p*NP-β-D-glucopyranoside, *p*NP-β-D-galactopyranoside, *p*NPR, *p*NPAF and *p*NPAP. The kinetic constant of the purified Xln-DT was measured by determining the initial rates at various *p*NPX ending concentrations (0.2, 0.4, 0.8, 1, 1.2, 1.4, 1.6, 1.8, 2, 3, 4, 5, 6 and 8 mM) under standard reaction conditions.

### Xylobiose degradation

The hydrolysis products from xylobiose by the purified Xln-DT were analyzed by TLC. Then, 10% xylobiose (wt/vol) was dissolved in 100 μL of citrate buffer solution (50 mM, pH 6.0) and incubated at 75 °C for 3 h with the addition of 1 U Xln-DT β-xylosidase. The reaction was terminated by heating in boiling water for 10 min. The hydrolysis products were analyzed by TLC on a silica gel plate (G254, Qingdao, China) using the solvent system containing n-butanol, ethanol and water (5:3:2, *v*/v/v) and were visualized using the orcinol sulfuric acid reagent.

### Enzymatic transformation of notoginsenoside R1 and ASI

For notoginsenoside R1 as a substrate, the reaction mixture contained 1 g/L notoginsenoside R1, 0.1 U Xln-DT and sodium phosphate buffer (50 mM, pH 6.0), and it was incubated at 75 °C for 30 min and then terminated by the addition of 400 μL methanol. For ASI as a substrate, the reaction mixture contained 1 g/L ASI, 0.5 U Xln-DT, 1 U Tpebg3 and sodium phosphate buffer (50 mM, pH 6.0), and it was incubated at 75 °C for 3 h and then terminated by the addition of 400 μL methanol. The methanol extract of this material was analyzed by HPLC.

The notoginsenoside R1 and ginsenoside Rg1 were analyzed using an Agilent HPLC 1260 system (USA) and a C18 column (4.6 × 250 mm; i.d., 5 μm; S.No. USNH017518, USA) with distilled water (A) and acetonitrile (B) as the mobile phase, with a gradient elution of 20% (B) from 0 to 3 min and 20-60% (B) from 3 to 28 min. The injection volume was 5 μL for each sample, the flow rate was 0.4 mL/min, and the detection was carried out by monitoring the absorbance at 203 nm. The ASI and CA were also analyzed using the same system and column described above, with distilled water (A) and acetonitrile (B) as the mobile phase, with A/B ratios of 40:60 from 0 to 16 min; the detection was performed by monitoring the ELSD. The injection volume was 10 μL for each sample, the flow rate was 1 mL/min, the gas flow rate was 2.1 L/min, and the ELSD drift tube temperature was 90 °C.

## Results

### Cloning and sequencing of the β-xylosidase gene *xln-DT*

**Fig. 1:**
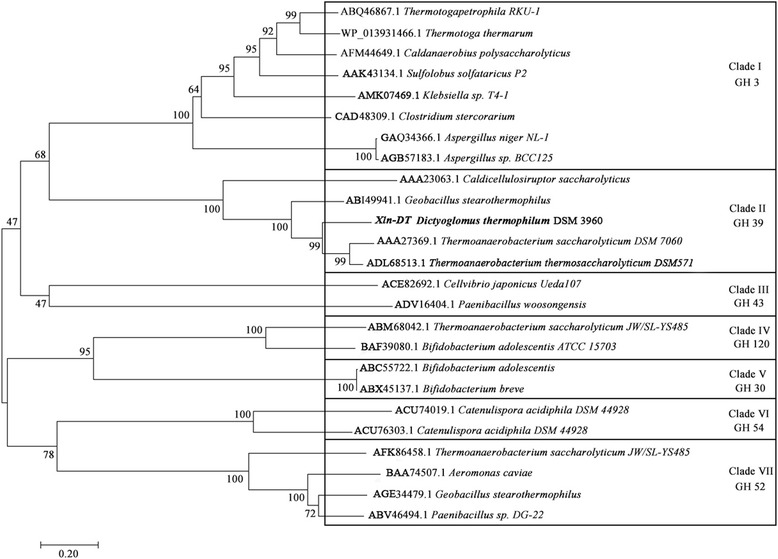
Neighbor-Joining (NJ) tree results from analysis of the Xln-DT β-xylosidase of 25 amino acid sequences. (Numbers on nodes correspond to percentage bootstrap values for 1000 replicates)

According to the analysis of the complete genome sequence of *Dictyoglomus thermophilum* DSM 3960, a protein with possible β-xylosidase activity (GenBank accession No. KX449145) encoded 502 amino acids with a full-length of 1509 bp and belonged to GH 39. To investigate the evolutionary relationship among the β-xylosidases, the phylogenetic trees were constructed by using the neighbor-joining method. The NJ trees revealed that there were seven clades, with each clade composed of a separated monophyletic group (Fig. 1). Clade I contained GH 3 β-xylosidases from bacteria, archaea and fungi, Clade II contained GH 39 β-xylosidases from several thermophilic bacteria, and Clade III to VII contained β-xylosidases that belonged to GH 43, 120, 30, 54 and 52, and were almost all from bacteria. Among the β-xylosidases from Clade II (GH 39), the members of the thermophilic genus D*ictyoglomus* had a distant relationship with *Thermoanaerobacterium*. Hence, it was posited that their enzymatic characterizations might be different. As is well known, GH 39 family β-xylosidases display a typical (α/β)_8_-barrel and perform hydrolysis in a two-stage reaction that consists of glycosylation and water-mediated deglycosylation steps. Based on the present database, it can be indicated that this protein could be a novel β-xylosidase that belongs to the GH 39 family.

The β-xylosidase gene *xln-DT* was cloned from the genomic DNA of *D.thermophilum* DSM 3960 and subcloned into the expression vector pET-20b to generate the plasmid pET-20b-*xln-DT*. Then the recombinant plasmid pET-20b-*xln-DT* was finally transformed into *E. coli* BL21 (DE3) and expressed with 0.1 mM IPTG. The activity of the recombinant β-xylosidase was approximately 2.3 U/mL in LB medium.

### Purification and characterization of recombinant Xln-DT β-xylosidase

**Fig. 2:**
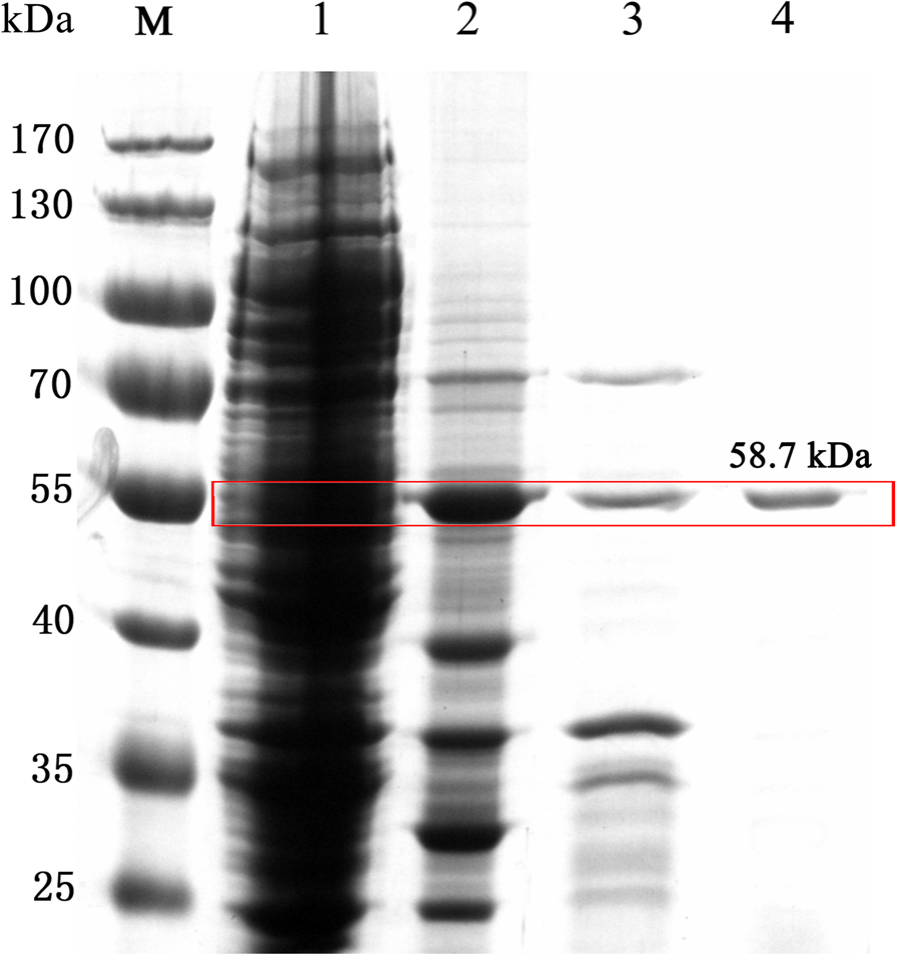
SDS-PAGE analysis of recombinant Xln-DT β-xylosidase expressed in *E.coli* BL21 (DE3). Lane M: protein marker; Lane 1: the crude extract of *E.coli* BL21 (DE3) harboring pET-20b; Lane 2: the crude extracts of Xln-DT β-xylosidase; Lane 3: the cell extracts after sonication were heat treated at 75 °C for 30 min; Lane 4: purified Xln-DT β-xylosidase by Ni-NTA resin affinity chromatography

For the biochemical characterization of the recombinant β-xylosidase, the crude β-xylosidase was purified by all the purification preparations. The crude β-xylosidase was given a single band on a 12% SDS-PAGE gel, and the MW of the enzyme was slightly more than 55 kDa without the other bands (Fig. 2), which was similar to the theoretical MW of the monomer (58.7 kDa). The specific activity of the purified Xln-DT was 2.9-fold higher than that of the crude enzyme with a purification yield of 52.6% (Table 1).

**Table 1:** Purification of recombinant protein Xln-DT

Purification step	Total activity(U)	Total protein(mg)	Specific activity (U/mg)	Yield (%)	Fold purification
Culture extract	539.1	187.2	2.88	100	1
Heat treatment^a^	392.3	67.3	5.83	72.8	2.02
Ni affinity chromatography	283.5	33.5	8.46	52.6	2.94

Substrate for Xln-DT was *p*-Nitrophenyl-β-D-xylopyranoside

^a^The cell extracts after sonication were heat treated at 75 °C for 30 min, and then cooled in an ice bath, centrifuged at 8000 g for 10 min at 4 °C and the supernatant was kept

**Fig. 3:**
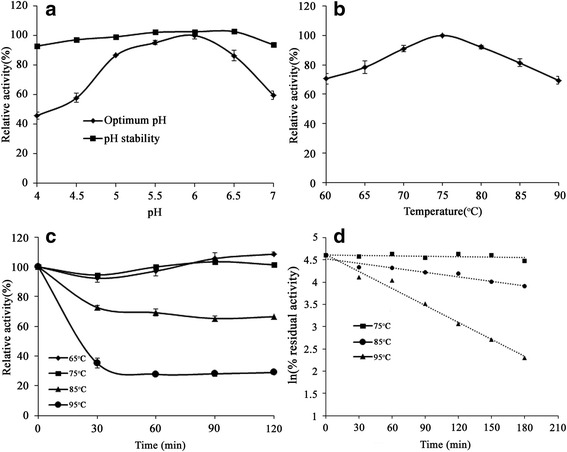
Characterization of recombinant Xln-DT β-xylosidase. **a** Effects of optimum pH and pH stability of the enzyme Xln-DT; **b** Effect of temperature on Xln-DT activity; **c** The thermostability of the enzyme Xln-DT. The residual activity was monitored while the enzyme was incubated at 65 °C (*filled diamonds*), 75 °C (*filled squares*), 85 °C (*flled triangles)* and 95 °C (*filled circles*). The maximum activity was defined as 100%; **d** The kinetic of thermal inactivation of the enzyme Xln-DT at different temperatures ranging from 75 °C to 95 °C for several time intervals (75 °C (*filled squares*), 85 °C (*filled circles*), 95 °C (*filled triangles*))

**Fig. 4:**
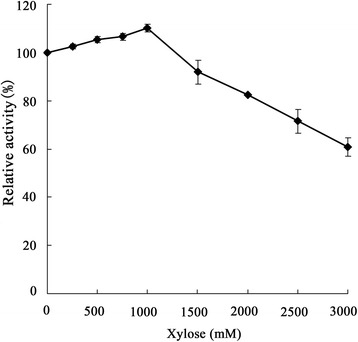
Effects of xylose on Xln-DT β-xylosidase activity

The enzymatic properties of the purified β-xylosidase Xln-DT were characterized. The optimal pH for Xln-DT was measured to be 6.0. The enzyme showed over 80% of the maximum activity at a pH from 5.0 to 6.5 (Fig. 3a). Moreover, the novel β-xylosidase showed good stability after being treated in sodium phosphate buffers at different pH levels for 24 h (Fig. 3a). The optimal temperature for Xln-DT was 75 °C. The enzyme showed more than 80% of the maximum activity at temperatures from 65 °C to 85 °C (Fig. 3b). Thermostability assays indicated that Xln-DT residual activity was above 60% of its initial activity at 65-85 °C when tested at pH 6.0 for 2 h (Fig. 3c). The kinetics of thermal inactivation were determined by incubating the enzyme at temperatures ranging from 75 °C to 95 °C for 3 h. As shown in Fig. 3d, the half-life of the recombinant β-xylosidase Xln-DT was approximately 29 h at 75 °C. At 95 °C, the half-life was approximately 1 h. In addition, Xln-DT had an unusually high tolerance to inhibition by xylose, up to 1 M xylose, which did not affect the Xln-DT activity, and 60% of the relative activity was reserved in 3 M xylose (Fig. 4).

The effects of the chemical reagents and metal ions on the enzyme activities of Xln-DT are shown in Table 2. The activity of Xln-DT was exceedingly inhibited by Hg^2+^ and Cu^2+^. Most of the other divalent metal cations had dissimilar effects on the activity of Xln-DT. In particular, the activity of Xln-DT, which was observably increased by Fe^2+^, Fe^3+^ and Mn^2+^, was similar to the alpha-mannosidase from the hen oviduct [41]. In addition, we incubated Xln-DT with Fe^2+^ at 75 °C for 3 h. The results showed that Fe^2+^ improved the thermostability of Xln-DT at 75 °C. The β-xylosidase activity of Xln-DT with 5 mM Fe^2+^ remained at over 300% after 3 h of incubation at 75 °C, while the Xln-DT without any metal ions only retained 80% activity. In addition, organic solvents such as methanol, ethanol and DMSO also affected the enzyme activity. The residual enzyme activity was over 100%, with 20% methanol or 10% ethanol. With the increasing concentrations of organic solvent, the enzyme activity was inhibited slowly.

**Table 2:** Effects of metal cations and reagents on the recombinant Xln-DT activity

Cation of reagent	Relative enzyme activity (%)	
	1 mM	5 mM
Contrast	100	100
PMSF	112.4	114.5
EDTA	94.3	90.5
Al^3+^	101.5	98.1
Cu^2+^	48.1	39.9
Zn^2+^	84.2	83.4
Ca^2+^	98.3	87.1
Fe^2+^	155.8	327.5
Fe^3+^	99.8	199.3
Na^+^	95.8	99.5
K^+^	101.3	101.4
Li^+^	92.3	91.1
Mg^2+^	102.8	97.1
NH_4_^+^	99.3	96.2
Mn^2+^	98.3	115.8
Ba^2+^	92.8	91.9
Hg^2+^	35.3	35.1
Co^2+^	95.8	89.6
Ni^2+^	108.2	89.2

Values shown were the mean of duplicate experiments, and the variation about the mean was below 5%

The substrate specificity of Xln-DT was measured by using 1.0 mM substrates (Table 3). The results illustrated that Xln-DT has a high activity toward *p*NPX and a lower activity against *p*NP-β-D-glucopyranoside, while no other glycosidase activity was detected against *p*NP-β-D-galactopyranoside, *p*NPAF, *p*NPAP and *p*NPR. The values of *K*_*m*_ and *V*_*max*_ were 1.66 mM and 78.46 U/mg, respectively, with *p*NPX as the substrate. The kinetic parameters were measured from Lineweaver-Burk plots. These results suggest that Xln-DT has highly specific activity toward residual xylose.

**Table 3:** Relative activity of recombinant Xln-DT towards various chromogenic substrates as measured by pNP release at 75 °C

Substrate^a^	Relative activity (mean% ± SD)^b^
*p*-Nitrophenyl-β-D-xylopyranoside	100 ± 2.01
*p-*Nitrophenyl-β-D-galactopyranoside	ND^c^
*p-*Nitrophenyl-α-L-arabinofuranoside	ND
*p*-Nitrophenyl-α-L-arabinopyranoside	ND
*p*-Nitrophenyl-α-L-rhamnopyranoside	ND
*p*-Nitrophenyl-β-D-glucopyranoside	23.1 ± 1.08

^a^Final concentration of each was 1.0 mM

^b^The relative enzyme activity aganist *p*-Nitrophenyl-β-D-xylopyranoside was assumed to be 100%

^c^Not determined, spectific activity is not determined by the analytical methods used in this study

### Xylobiose hydrolyzation of Xln-DT

**Fig. 5:**
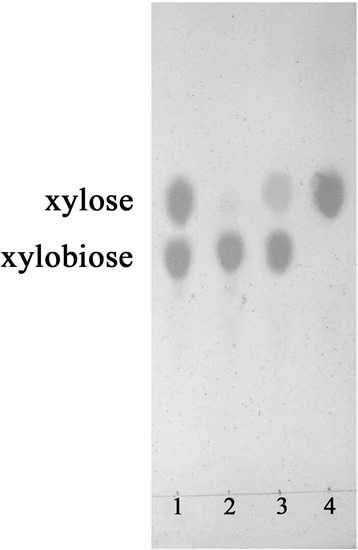
TLC analysis of xylobiose hydrolyzed by Xln-DT. Lane1: Standards; Lane2: Xylobiose before reaction; Lane3: Xylobiose after reaction for 30 min; Lane4: Xylobiose after reaction for 3 h

Xylobiose was incubated with purified Xln-DT β-xylosidase and examined using TLC (Fig. 5). After 3 h, all the xylobiose tested was completely degraded to xylose. This result indicates that this cloned enzyme is a true β-xylosidase with hydrolytic activity against short xylooligosaccharides.

### Biotransformation of notoginsenoside R1 and ASI by Xln-DT

**Fig. 6:**
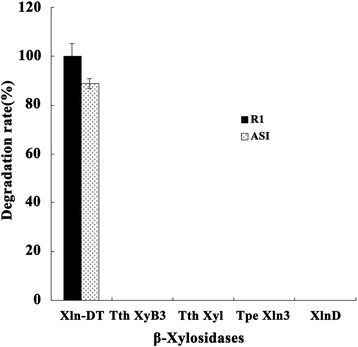
Comparison of enzymatic conversion efficiency of notoginsenoside R1 and ASI to ginsenoside Rg1 and CA by β-xylosidases from different sources. (Xln-DT was from *Dictyoglomus thermophilum* DSM3960*,* Tth XyB3 was from *Thermotoga thermarum* DSM 5069, Tth Xyl was from *Thermoanaerobacterium thermosaccharolyticum* DSM 571, Tpe Xln3 was from *Thermotoga petrophila* DSM 13995, XlnD was from *Aspergillus niger NL-1*)

The biotransformation pathway from notoginsenoside R1 to ginsenoside Rg1 cleaved the outer xylose moiety at position C-6 of notoginsenoside R1 via β-xylosidase (Additional file 1: Figure S1). The biotransformation pathway from ASI to CA cleaved the outer glucose moiety at position C-6 and the xylose moiety at position C-3 of ASI via β-glucosidase and β-xylosidase, respectively (Additional file 1: Figure S2). As shown in Fig. 6, three GH 3 family β-xylosidases Tth XyB3, Tpe Xln3 and XlnD from *Thermotoga thermarum* DSM 5069, *Thermotoga petrophila* DSM 13995 and *Aspergillus niger NL-1,* respectively, one GH 120 family β-xylosidase Tth Xyl from *Thermoanaerobacterium thermosaccharolyticum* DSM 571 and Xln-DT (GH 39) were selected to biotransform notoginsenoside R1 and ASI. Only the β-xylosidase Xln-DT from GH 39 family had 100 and 88.9% of the biotransformation rate for notoginsenoside R1 and ASI (Additional file 1: Figures S3 and S4). A total of 1 g/L of notoginsenoside R1 was converted into 0.86 g/L of ginsenoside Rg1 after 30 min, with a corresponding molar conversion yield of 100% in total. A total of 1 g/L of ASI was converted into 0.36 g/L of CA after 3 h, with a corresponding molar conversion yield of 88.9% in total.

## Discussion

According to the database, more than 130 families were classified as glycoside hydrolases. Through the GenBank blast program, β-xylosidase Xln-DT belonged to the GH 39 family. This enzyme has the highest sequence similarity of 87% with β-xylosidase from *Caldicellulosiruptor saccharolyticus* (GenBank accession No. WP_049754849.1) and shares a 72% similarity with the β-xylosidase from *Thermoanaerobacterium thermosaccharolyticum* (GenBank accession No. WP_013297482.1). The phylogenetic analysis showed that Xln-DT had a distant relationship with other β-xylosidases belonging to the GH 39 family. This paper is the first report on the purification and biochemical characterization of Xln-DT β-xylosidase from *Dictyoglomus thermophilum.*

Generally, fungal β-xylosidases have an optimal pH under 5.0, while bacterial β-xylosidases are active up to pH 7.0. In this paper, the optimum pH of the recombinant Xln-DT from *Dictyoglomus thermophilum* was found to be nearly neutral (pH 6.0), which was similar to the β-xylosidase from *Paecilomyces thermophila* and GH 39 β-xylosidase from *Caulobacter crescentus* [26, 42]. The enzymatic characterization indicated that Xln-DT had a highly optimal temperature at 75 °C, which was higher than that observed for the β-xylosidases from *Aspergillus niger*, *Sporotrichum thermophile* and *Caulobacter crescentus* [43, 44]. Moreover, Xln-DT had a preferable temperature tolerance under 85 °C, which was similar to the thermostable GH 3 β-xylosidase from *Thermotoga thermarum* [36]. In practical applications, an enzyme with high heat resistance is desirable preferentially because of its longer half-life (29 h for 75 °C) implying lower a consumption of the enzyme. The recombinant β-xylosidase has satisfactory pH and temperature stability, which is the favorable factor for the potential applications of the enzyme, such as in the biotransformation field.

As is well known, highly xylose-tolerant β-xylosidase has great potential in biochemical application fields because xylose is a strong inhibitor of β-xylosidase. Unfortunately, the majority of microbial β-xylosidases are sensitive to xylose. Table 4 summarizes the xylose-resistant ability from different organisms. Most β-xylosidases from fungi, such as *Neurospora crassa* and *Aspergillus niger B 03*, display a *K*_*i*_ for xylose below 2 mM. The bacterial β-xylosidases from *Thermoanaerobacterium saccharolyticum*, *Thermotoga thermarum* and *Scytalidium thermophilum* were insensitive to inhibition, ranging from 200 mM to 1000 mM xylose*.* In this study, it was surprising that Xln-DT had a very prominent tolerance to inhibition by xylose. There was no inhibition at 1 M xylose, and there was a 40% inhibition at 3 M xylose. Among the previously reported β-xylosidases, the *K*_*i*_ for xylose of Xln-DT β-xylosidase was the highest among those reported. At present, there is no literature to analyze the xylose-tolerance mechanism of β-xylosidase, and only a few studies have reported the mechanism of glucose tolerance and stimulation of glucosidases [45]. The Xln-DT β-xylosidase with high xylose tolerance and stimulation suggested that some active sites at the entrance and middle of the substrate channel modulate the effects of xylose, which give the GH 39 β-xylosidase a more prominent application potential in industries. The purified enzyme activity was measured by the presence of the tested ions. A 1 mM (or 5 mM) concentration of Cu^2+^ and Hg^2+^ inhibited the enzyme activity significantly, which was relatively common for this enzyme from *Paecilomyces thermophila* [46]. The catalytic efficiency of the reaction system with Fe^2+^ and Fe^3+^ added at a 5 mM final concentration was nearly 3.2- and 1.9- times higher, respectively, than enzymes without pre-incubation of the metal ions, indicating that the Fe ion could be the element of the catalytic active center and a stable factor for the structure of the aim protein. A 1 mM or 5 mM concentration of EDTA had no significant effect on the enzyme activity, which suggests that the chelating agent EDTA did not affect the β-xylosidase activity and was not essential for the catalytic activity of this enzyme. The organic modulator, PMSF (1 mM or 5 mM), had slightly promoting role on enzyme activity. Moreover, Fe^2+^ and Fe^3+^ improved the thermostability of Xln-DT at 75 °C for 3 h, which indicated that this protein may have a metal-ion binding loop. By binding metal ions such as Fe^2+^ and Fe^3+^, the loop can keep the thermal stability of the protein structure. Since organic solvents have been used for solubilizing water-insoluble substrates in enzymatic reactions, the enzyme with organic solvent resistance has a higher application value. As shown in Table 5, low concentrations of organic solvents, such as methanol, ethanol and DMSO, promoted enzyme activity, which was similar to the β-glucosidase from *Thermotoga petrophila*. This enzyme seemed to be tolerant to methanol and ethanol, since it was almost uninhibited in 20% of methanol and retained over 80% of its residual activity in the presence of 30% methanol. This level of tolerance was higher than that previously reported of β-xylosidases from *Geobacillus thermodenitrificans* [35]. The results indicated that the enzyme could be used in industrial applications in the presence of some organic solvents.

**Table 4:** Xylose resistant ability of Xln-DT and β-xylosidase from other microorganisms

No.	Organism	Xylose resistant ability	Reference
1	*Dictyoglomus thermophilum*	60% of its activity was retained in the presence of 3 M xylose	This study
2	*Paecilomyces thermophila*	a *K*_*i*_ value of 139 mM	[46]
3	*Aspergillus niger B 03*	a *K*_*i*_ value of 1.857 mM	[44]
4	*Thermotoga thermarum*	a *K*_*i*_ value of 1000 mM	[36]
5	*Thermoanaerobacterium saccharolyticum*	70% of its activity was retained in the presence of 200 mM xylose	[48]
6	*Scytalidium thermophilum*	Xylose inhibits most β-xylosidase at concentrations up to 200 mM	[47]
7	*Neurospora crassa*	a *K*_*i*_ value of 1.72 mM	[30]
8	*Humicola insolens*	*K*_*i*_ values for Xyl43A and Xyl43B were 79 and 292 mM, respectively.	[49]
9	*Aureobasidium pullulans CBS 135684*	a *K*_*i*_ value of 18 mM	[50]

**Table 5:** The effects of organic solvent for the recombinant Xln-DT activity

Final concentration of organic solvent (%)	Relative enzyme activity (%)		
	Methanol	Ethanol	DMSO
0	100	100	100
5	110.5	115.6	90.1
10	118.7	100.3	82.2
15	118.5	87.6	68.6
20	113.7	76.4	63.9
25	88.5	58.6	56.5
30	63.4	41.1	36.2

Values shown were the mean of duplicate experiments, and the variation about the mean was below 5%

β-xylosidase is an exoglycosidase with the ability to degrade the non-reducing ends of xylooligosaccharides into xylose, which is one kind of hemicellulolytic enzyme. Compared with the other β-xylosidases, Xln-DT possessed higher efficiency in xylobiose hydrolysis. Reported *K*_*m*_ values of other β-xylosidases for bacterial enzymes ranged from 0.018 mM to 10 mM, while the *K*_*m*_ values were normally over 1 mM for fungal β-xylosidases. The *K*_*m*_ and *V*_*max*_ values of Xln-DT for *p*NPX were 1.66 mM and 78.46 U/mg, respectively, which were higher than the β-xylosidase from *S.thermophilum* with 65 U/mg [47]. Moreover, the capacity of the β-xylosidase to hydrolyze xylobiose was studied by using 10% xylobiose incubated with the purified enzyme at 75 °C. After 3 h, the xylobiose was completely hydrolyzed into xylose, which revealed the obvious ability to convert the XOs into xylose.

As is well known, conventional β-xylosidases are frequently used to hydrolyze the end bone of β-1, 4-linked xylopyranosyl. However, for notoginsenoside R1 and ASI, it was the 1, 2-glycosidic bond. According to this paper, only Xln-DT could cleave the outer xylose moiety at position C-6 of the notoginsenoside R1 and ASI, which indicated that it had highly specificity in biotransforming notoginsenoside R1 and ASI to ginsenoside Rg1 and CA, without attacking the other glycosidic linkages. Although ginsenoside Rg1 and CA could be prepared by traditional methods, the yield was low, and it was time and money consuming. In addition, ginsenoside Rg1 and CA were accompanied with some by-products by traditional methods, which greatly impede later purification. Taken together, as a clean and green technology, biotransformation with specific enzymes showed the highest efficiency and specificity. All these results suggest that this recombinant Xln-DT has great potential for industrial applications, especially for bio-transformation to produce natural medicine.

## Conclusions

In this paper, a novel β-xylosidase, Xln-DT, from *Dictyoglomus thermophilum* DSM 3960 was cloned and expressed in *E. coli* BL21. The phylogenetic analysis showed that Xln-DT had a distant relationship with other β-xylosidases belonging to the GH 39 family. The enzymatic characterization displayed that Xln-DT had a high optimal temperature and a partially neutral pH, and the thermostability was improved by the ion Fe. Most importantly, there was no inhibition at 1 M xylose, and there was a 40% inhibition at 3 M xylose. Compared with the other β-xylosidases, Xln-DT possessed higher efficiency in xylobiose hydrolysis and had efficiency for biotransforming notoginsenoside R1 and ASI by removing the 1, 2 glycosidic bond linked to the C-6 and C-3 carbons. This study indicates that recombinant Xln-DT would be suitable for producing natural medicine, such as ginsenoside Rg1 and CA.

## Additional file

Additional file 1: **Figure S1.** Biotransformation pathway for production of ginsenoside Rg1 from notoginsenoside R1. **Figure S2.** Biotransformation pathway for production of CA from ASI. **Figure S3.** HPLC analysis of notoginsenoside R1 hydrolysis by Xln-DT. (a) Standards of notoginsenoside R1 and ginsenoside Rg1; (b-d) notoginsenoside R1 (1 g/L) incubated with Xln-DT for 0, 5 and 30 min, respectively. **Figure S4.** HPLC analysis of ASI hydrolysis by Xln-DT. (a) Standards of ASI and CA; (b-d) ASI (1 g/L) incubated with Xln-DT and Tpebg3 for 0, 1 and 3 h, respectively. (DOC 5520 kb)

## Abbreviations

ASI: Astragaloside IV
CA: Cycloastragenol
*E.coli*: *Escherichia coli*
EDTA: Ethylenediaminetetraacetic acid
ELSD: Evaporative Light Scattering Detector
GH: Glycosyl hydrolase
HPLC: High performance liquid chromatography
IPTG: Isopropyl-β-D-thiogalactopyranoside
LB: Luria-Bertani
MW: Molecular weight
NJ: Neighbor-joining
PCR: Polymerase chain reaction
pNPAF: p-nitrophenyl-α-L-arabinofuranoside
pNPAP: p-nitrophenyl-α-L-arabinopyranoside
pNPR: p-nitrophenyl-α-L-rhamnopyranoside
pNPX: p-nitrophenyl-β-D-xylopyranoside
QC: Quality control
SDS-PAGE: Sodium dodecyl sulfate polyacrylamide gel electrophoresis
TLC: Thin-layer chromatography
XOs: Xylooligosaccharides
