# Supplementary material for: Characterization of a novel thermostable and xylose-tolerant GH 39 β-xylosidase from Dictyoglomus thermophilum
Source: BMC Biotechnol. 2018 May 21;18:29. doi: 10.1186/s12896-018-0440-3 (PMC5963010; doi:10.1186/s12896-018-0440-3)
Supplement: Supplementary file 1 — Figure S1. Biotransformation pathway for production of ginsenoside Rg1 from notoginsenoside R1. Figure S2. Biotransformation pathway for production of CA from ASI. Figure S3. HPLC analysis of notoginsenoside R1 hydrolysis by Xln-DT. (a) Standards of notoginsenoside R1 and ginsenoside Rg1; (b-d) notoginsenoside R1 (1 g/L) incubated with Xln-DT for 0, 5 and 30 min, respectively. Figure S4. HPLC analysis of ASI hydrolysis by Xln-DT. (a) Standards of ASI and CA; (b-d) ASI (1 g/L) incubated with Xln-DT and Tpebg3 for 0, 1 and 3 h, respectively. (DOC 5520 kb) [file 12896_2018_440_MOESM1_ESM.doc]

**BMC Biotechnology**

**Characterization of a novel thermostable and xylose-tolerant GH39** **β-xylosidase from** ***Dictyoglomus thermophilum***

Qi Li^1,2,3^, Tao Wu^2^, Zhipeng Qi^2^, Linguo Zhao^1,2,3*^, Jianjun Pei^2,3^, Feng Tang^4^

^1^Co-Innovation Center for Sustainable Forestry in Southern China, Nanjing Forestry University, 159 Long Pan Road, Nanjing 210037, China

^2^College of Chemical Engineering, Nanjing Forestry University, 159 Long Pan Road, Nanjing 210037, China

^3^Jiangsu Key Lab for the Chemistry & Utilization of Agricultural and Forest Biomass, 159 Long Pan Road, Nanjing 210037, China

^4^International Centre for Bamboo and Rattan, 8 Fu Tong East Street, Beijing 100714, China

^*^Corresponding authors for Linguo Zhao at College of Chemical Engineering, Nanjing Forestry University, Nanjing, 210037, China.

Phone: +86-025-85427962.

Fax:+86-025-85427300.

E-mail:njfu2304@163.com.

**Figure S1** Biotransformation pathway for production of ginsenoside Rg1 from notoginsenoside R1.

**
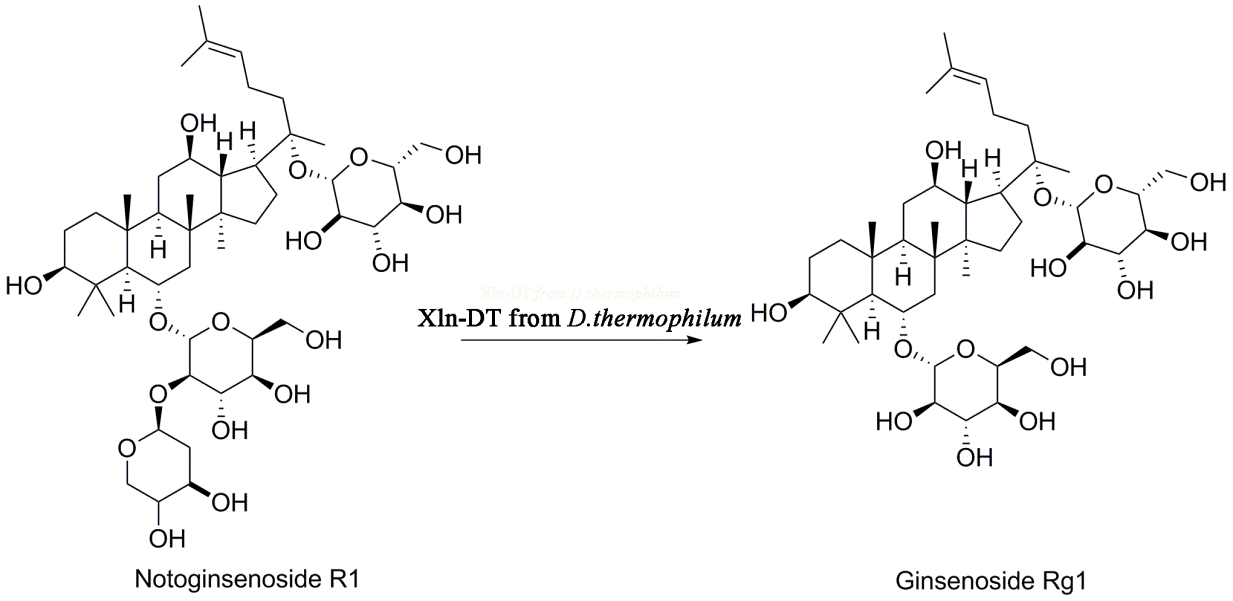
**

**Figure S2** Biotransformation pathway for production of CA from ASI.

**
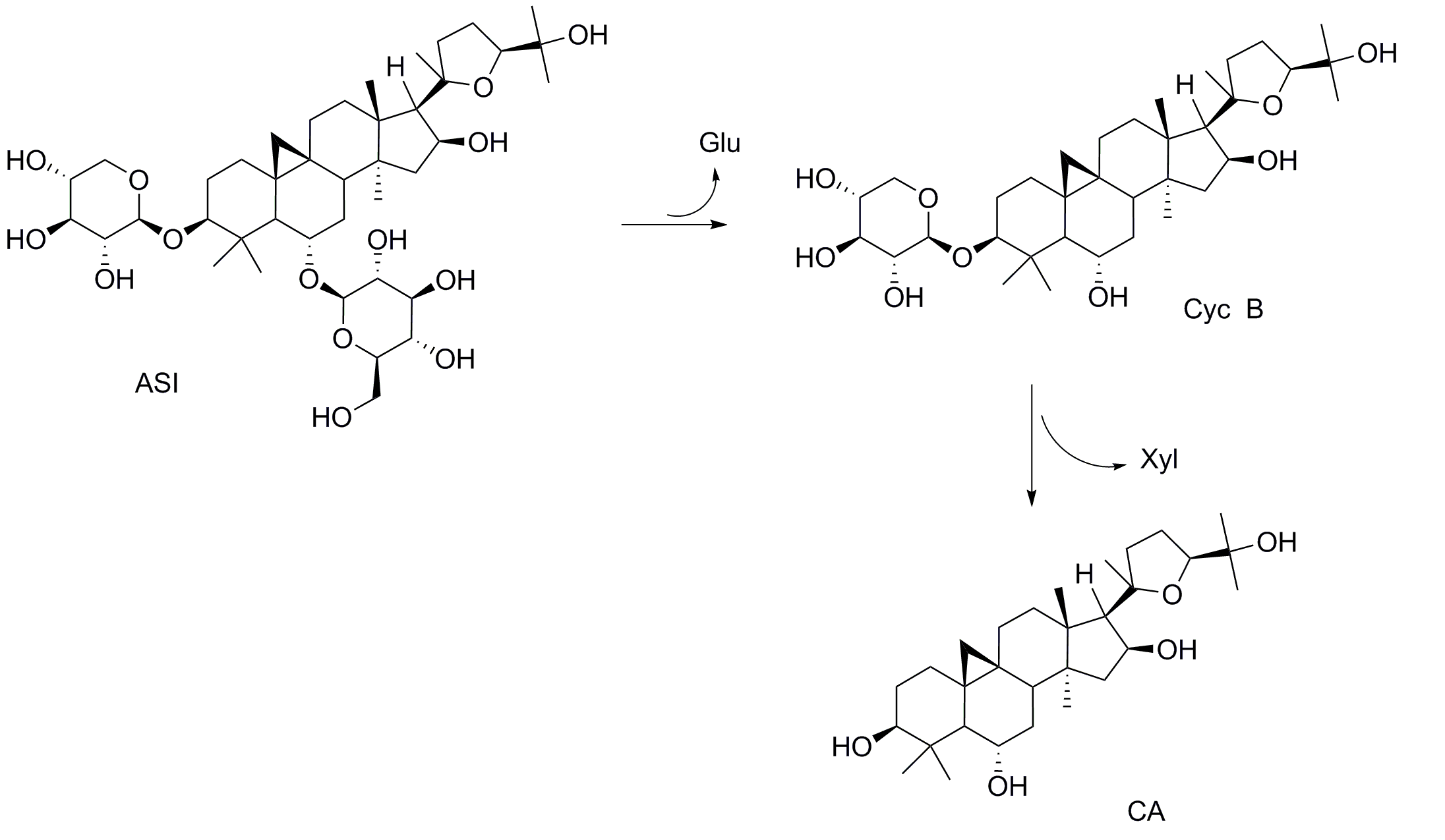
**

**Figure S3** HPLC analysis of notoginsenoside R1 hydrolysis by Xln-DT. (a) Standards of notoginsenoside R1 and ginsenoside Rg1; (b-d) notoginsenoside R1 (1 g/L) incubated with Xln-DT for 0, 5 and 30 min, respectively.

**

**

**Figure S4** HPLC analysis of ASI hydrolysis by Xln-DT. (a) Standards of ASI and CA; (b-d) ASI (1 g/L) incubated with Xln-DT and Tpebg3 for 0, 1 and 3 h, respectively.

**
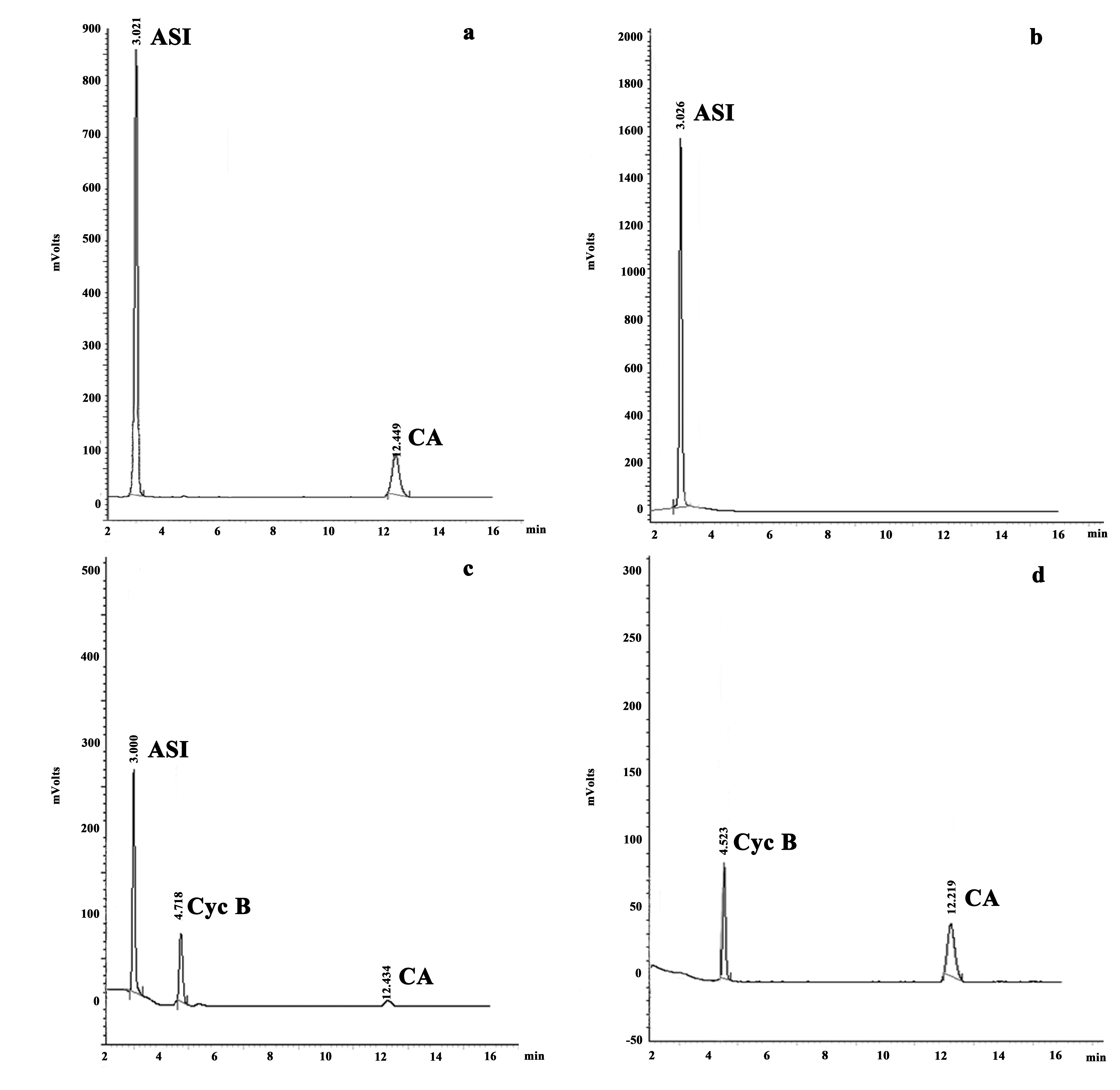
**
